# Design engineering a walking robotic manipulator for in-space assembly missions

**DOI:** 10.3389/frobt.2022.995813

**Published:** 2022-10-14

**Authors:** Manu Harikrishnan Nair, Mini Chakravarthini Rai, Mithun Poozhiyil

**Affiliations:** Lincoln Centre for Autonomous Systems, University of Lincoln, Lincoln, United Kingdom

**Keywords:** in-space assembly, large aperture space telescope, mission requirements, assembly challenges, walking robotic manipulator, design engineering, finite element analysis

## Abstract

In-Space Services aim to introduce sustainable futuristic technology to support the current and growing orbital ecosystem. As the scale of space missions grows, there is a need for more extensive infrastructures in orbit. In-Space Assembly missions would hold one of the key responsibilities in meeting the increasing demand. In the forthcoming decades, newer infrastructures in the Earth’s orbits, which are much more advanced than the International Space Station are needed for *in-situ* manufacturing, servicing, and astronomical and observational stations. The prospect of in-orbit commissioning a Large Aperture Space Telescope (LAST) has fuelled scientific and commercial interests in deep-space astronomy and Earth Observation. However, the *in-situ* assembly of such large-scale, high-value assets in extreme environments, like space, is highly challenging and requires advanced robotic solutions. This paper introduces an innovative dexterous walking robotic system for in-orbit assembly missions and considers the Large Aperture Space Telescope system with an aperture of 25 m as the use case. The top-level assembly requirements are identified with a deep insight into the critical functionalities and challenges to overcome while assembling the modular LAST. The design and sizing of an End-over-end Walking Robot (E-Walker) are discussed based on the design of the LAST and the specifications of the spacecraft platform. The E-Walker’s detailed design engineering includes the structural finite element analysis results for space and earth-analogue design and the corresponding actuator selection methods. Results of the modal analysis demonstrate the deflections in the E-Walker links and end-effector in the open-loop due to the extremities present in the space environment. The design and structural analysis of E-Walker’s scaled-down prototype is also presented to showcase its feasibility in supporting both in-orbit and terrestrial activities requiring robotic capabilities over an enhanced workspace. Further, the mission concept of operations is presented based on two E-Walkers that carry out the assembly of the mirror modules. The mission discussed was shortlisted after conducting an extensive trade-off study in the literature. Simulated results prove the dual E-Walker robotic system’s efficacy for accomplishing complex *in-situ* assembly operations through task-sharing.

## Introduction

The evolution of robotics and artificial intelligence has revolutionised state-of-the-art space systems. Robotics, Automation and Autonomous Systems (RAAS) solutions have helped the space community conduct ground-breaking research in various planetary missions and on the International Space Station (ISS). Building upon these substantial achievements, the next few decades would unfold new chapters in orbital missions. Over the years, Extra-Vehicular Activities (EVA) have proven beneficial for servicing and maintenance missions on the ISS (Culbertson 2003). The installation of corrective optics on the Hubble Space Telescope (HST) is another explicit example of the success of EVA (Burrows 1991). However, the unfavourable space environment constantly risks EVA operations (Thirsk et al., 2009; Fazekas 2020). With future missions involving large-scale high-value infrastructures, assembly and maintenance would be much beyond human capabilities, requiring autonomous robots. Advancements in RAAS have resulted in a paradigm shift in space exploration as it would facilitate a multitude of In-Space services. In support of the plans to be a dominant candidate in the In Orbit Services and Manufacturing (IOSM) market, the UK Space Agency, along with multiple enterprises, are in plans to invest in relevant futuristic space technologies (Catapult 2020). In-Space services can facilitate numerous missions such as manufacturing, assembly, servicing, debris removal, astronomy, and Earth Observation (EO), to list a few (Alondra Nelson et al., 2022). Amongst the innumerable tasks which In-Space services can expedite, this paper focuses on assembling a Large Aperture Space Telescope (LAST) in orbit. LAST would promote a series of astronomical and EO missions (Baiocchi and Stahl 2009, Catapult 2020).

The move towards LAST commenced ever since the successful launch of the HST. Launched in 1990, the HST with a 2.4m monolithic Primary Mirror (PM), became the ‘eye in the sky’ for any astronomer. It has helped mankind pin down the age of our cosmos, prove the existence of supermassive black holes and other numerous contributions (Hubble 2020). Thereafter, its successor, the James Webb Space Telescope (JWST) mission, was launched in December 2021. The JWST has a folded-wing design to incorporate the 6.5m PM, with capabilities to visualise a hundred million years after the Big Bang (Lockwood Optics 2020). As understood, there exists a constant scientific and commercial clamour to upgrade the telescope sizing for a better resolution and signal-to-noise ratio. Addressing these requirements, the space community is moving towards deploying telescopes with a much larger aperture in orbit. Assembling a LAST on the ground is not practical given the limited fairing size of current and planned launch vehicles (Nelson, Mast and Chanan 2013; Garzón et al., 2017). Much like the trend for planned futuristic space designs, LAST would facilitate a modular design approach for the mirror units to fit well within the fairing limits of the launcher. The modules of the LAST system are to be autonomously assembled in orbit using RAAS. Existing literature provides conceptual models of various approaches to carry out *in-situ* assembly missions of LAST. These include a hexbot, robots inspired by the Special Purpose Dexterous Manipulator, a fixed-to-base robotic manipulator concept and mobile robotic manipulators, to enlist a few (Oegerle et al., 2006; Lee et al., 2016; Nanjangud et al., 2019; Rognant et al., 2019). However, a wide knowledge gap exists in design engineering autonomous space robotic systems. Therefore, it is of utmost importance to shift from concepts to simulations and eventually realise large-scale robotic assembly in orbit. Previous studies presented a five Degrees-of-Freedom (DoF) End-Over-End Walking Robot (E-Walker) as a potential candidate for efficiently assembling a LAST in-orbit (Nair et al., 2020a; Nair et al., 2020b; Nair et al., 2020c). The initial design, kinematic and dynamical modelling of the E-Walker integrated with an industrially proven Proportional-Integral-Derivative Computed-Torque Controller (PID-CTC) to successfully track the joint parameters within its limits was discussed in (Nair et al., 2020a). A detailed feasibility analysis on a series of mission concepts and a potential mission concept was presented in (Nair et al., 2020b; Nair et al., 2020c).

This paper presents an updated design of the E-Walker for efficiently assembling a 25 m LAST in orbit. Initially, this paper elicits the top-level assembly requirements of LAST, the robotic system and the spacecraft platform. To meet the mission requirements, a seven Degrees-of-Freedom (DoF) fully dexterous E-Walker is introduced. The E-Walker draws inspiration from the Canadarm2 and the European Robotic Arm (ERA) on the ISS. The two links of the Canadarm2 are separated by a joint offset in the elbow, making it complicated for the robot arm to walk around connector ports placed in a straight line. On the other hand, the ERA has both its links attached to the side of its elbow joint, limiting the complete rotation of its the elbow joint like the Canadarm2 to an end-to-end motion. In comparison to Canadarm2 and ERA, the E-Walker has both its links in line about its elbow joint, allowing it to walk end-over-end like Canadarm2 without an offset. Either end of the E-Walker is equipped with a Latching End-Effector (LEE) to latch onto the static connector ports on LAST and the spacecraft platform. The proposed E-Walker’s redundant design and mobility features offer access to a much larger workspace than conventional fixed-to-base robotic manipulators. Also, the LAST design presented can be considered as a baseline for futuristic LAST designs, which can be scaled up for PMs up to 100 m aperture.

**FIGURE 1:**
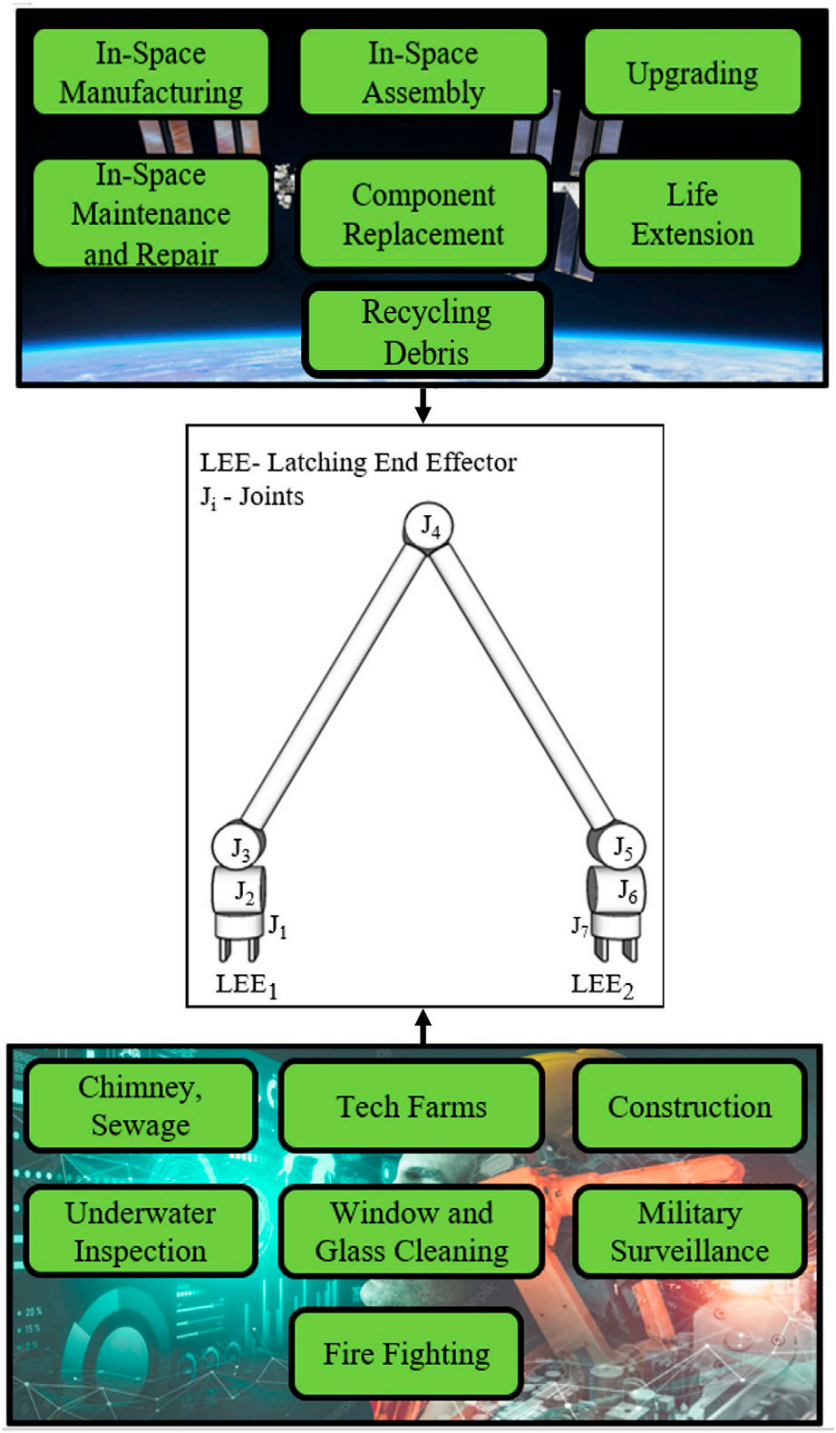
E-Walker applications and configuration.

An in-depth design engineering exercise has been carried out for the E-Walker according to the system and mission requirements for 1) optimising the link and LEE parameters 2) actuator selection. Initially, different link shapes of a given link length were compared to analyse the mass and area moment of inertia. Thereafter, to evaluate deflection, stress and strain, and open-loop performance under external disturbances in orbit, Finite Element Analysis (FEA) was carried out for the E-Walker components through Static Structural Analysis (SSA) and Modal analysis. The torque requirements for joint actuators were obtained using E-Walker’s dynamic equation formulation, which eventually helped in selecting the electrical actuators for the system. Additionally, a scaled-down prototype for the Earth-analogue testing is introduced, which underwent a similar FEA analysis. The E-Walker prototyping work is now in progress at the University of Lincoln; therefore, the experimental verification and validation will be published separately. Figure 1 presents an overview of various applications of the E-Walker along with its configuration. Finally, this paper discusses a potential mission concept of operations (ConOps) to carry out a sequential assembly of the 25 m LAST. The presented mission concept is chosen based on an extensive feasibility study carried out in (Nair et al., 2020b). The ConOps utilise two E-Walkers to assemble the PM, showcasing the extensive cooperative task-sharing capabilities of a dual robotic system, much required in futuristic robotic space missions.

The rest of the paper is organised into five sections. *Top-level assembly requirements* discusses the LAST mission’s top-level assembly requirements, including the robot and spacecraft system requirements. The Space grade E-Walker’s extensive design engineering is presented in *End-over-end walking robot*, where the full-scale system is optimised and analysed using SSA and Modal analysis under microgravity conditions. *E-walker scaled-down Earth analogue design* introduces the scaled-down Earth-analogue E-Walker prototype design. *Mission concept of operations* discusses a mission concept involving a dual E-Walker system to carry out the assembly. A flowchart is presented to depict the execution of this mission. In *Conclusion and future research*, the main inferences are covered, including an insight into planned experimental testing and further research.

## Top-level assembly requirements

The top-level system architecture of the 25m LAST mission comprises the robotic system (E-Walker), the 25m LAST and the spacecraft platform. The spacecraft platform is a composite system inclusive of the B_sc_, S_sc_ and truss modules. Throughout the paper, it is assumed that the components of the spacecraft platform remain docked. To carry out the assembly tasks in orbit, each system should meet certain requirements for a safe and precise assembly. The E-Walker has to meet certain mission-oriented goals to carry out the assembly of the 25m LAST, i.e., in terms of mobility, and pick and place operations. Table 1 presents the top-level requirements of the E-Walker.

**TABLE 1:** Top-level requirement list for E-Walker.

Sl. No	Requirements
$R_{1}$	The E-Walker should be able to assemble the telescope in space. Post assembly, it should be able to service any mirror module. To aid this requirement, the following specifications are recommended
	• Full dexterity using a 7DoF E-Walker
	• Total length of the E-Walker should enable it to traverse across the spacecraft platform *via* connector ports
	• Two LEEs at each end to connect to the connector ports
	• Select actuators to provide sufficient torque to lift a payload of one PMS.
	• Material selection should take into consideration the extremities of the space environment
$R_{2}$	The E-Walker should carry out precise and delicate handling of the mirror modules
$R_{3}$	The mobility feature of the E-Walker should be utilised to enhance its workspace
$R_{4}$	The E-Walker should avoid any collision with the spacecraft platform and with the mirror modules. If multiple E-Walkers are used, the path-planning algorithm should also consider collision avoidance with each E-Walker
$R_{5}$	Once the telescope is operational, the E-Walker shall not obstruct its field of view

### 25m PM requirements

**FIGURE 2:**
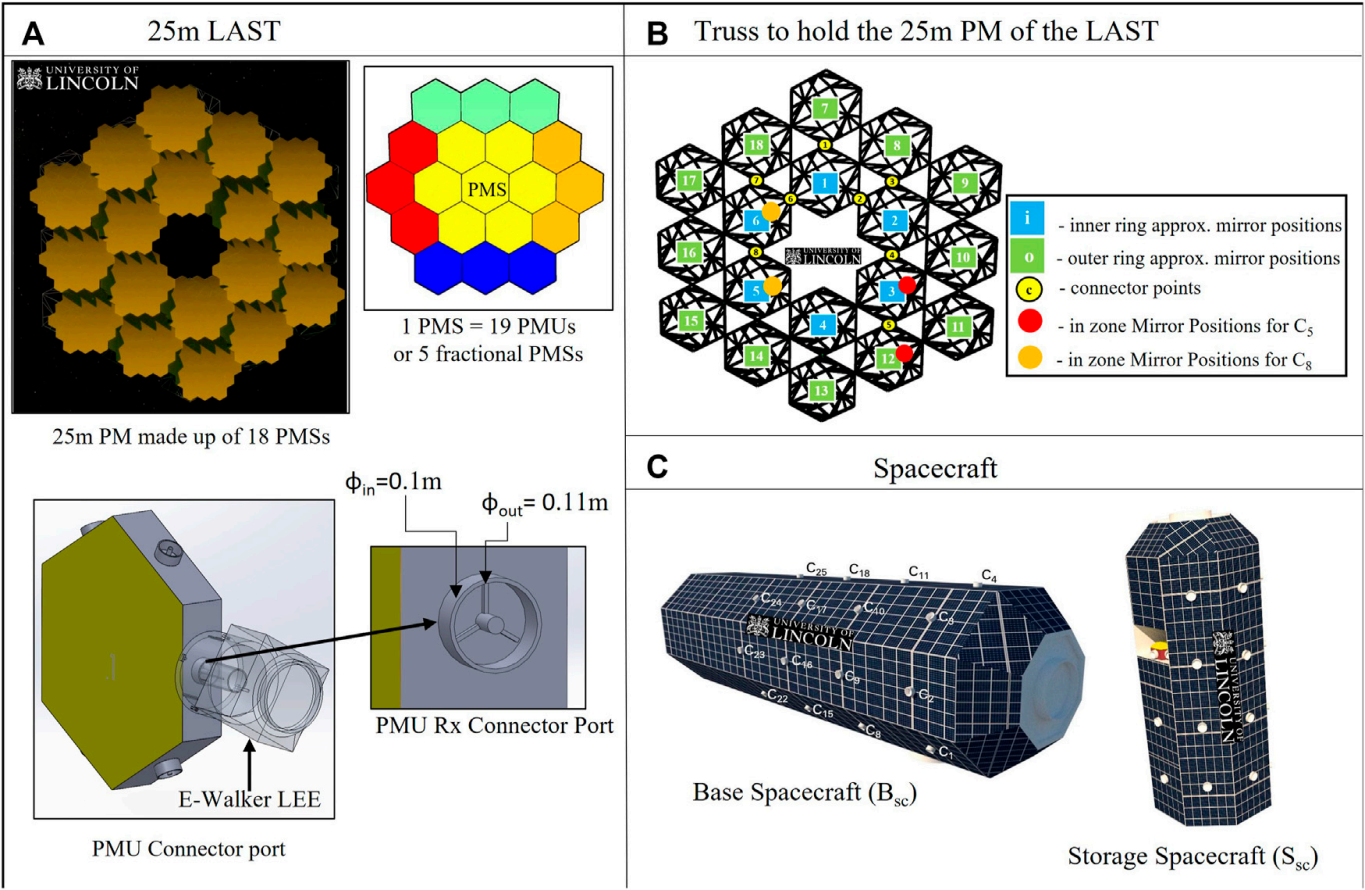
Top-level mission architecture **(A)** 25m last **(B)** truss; **(C)** B_sc_ and S_sc_.

**FIGURE 3:**
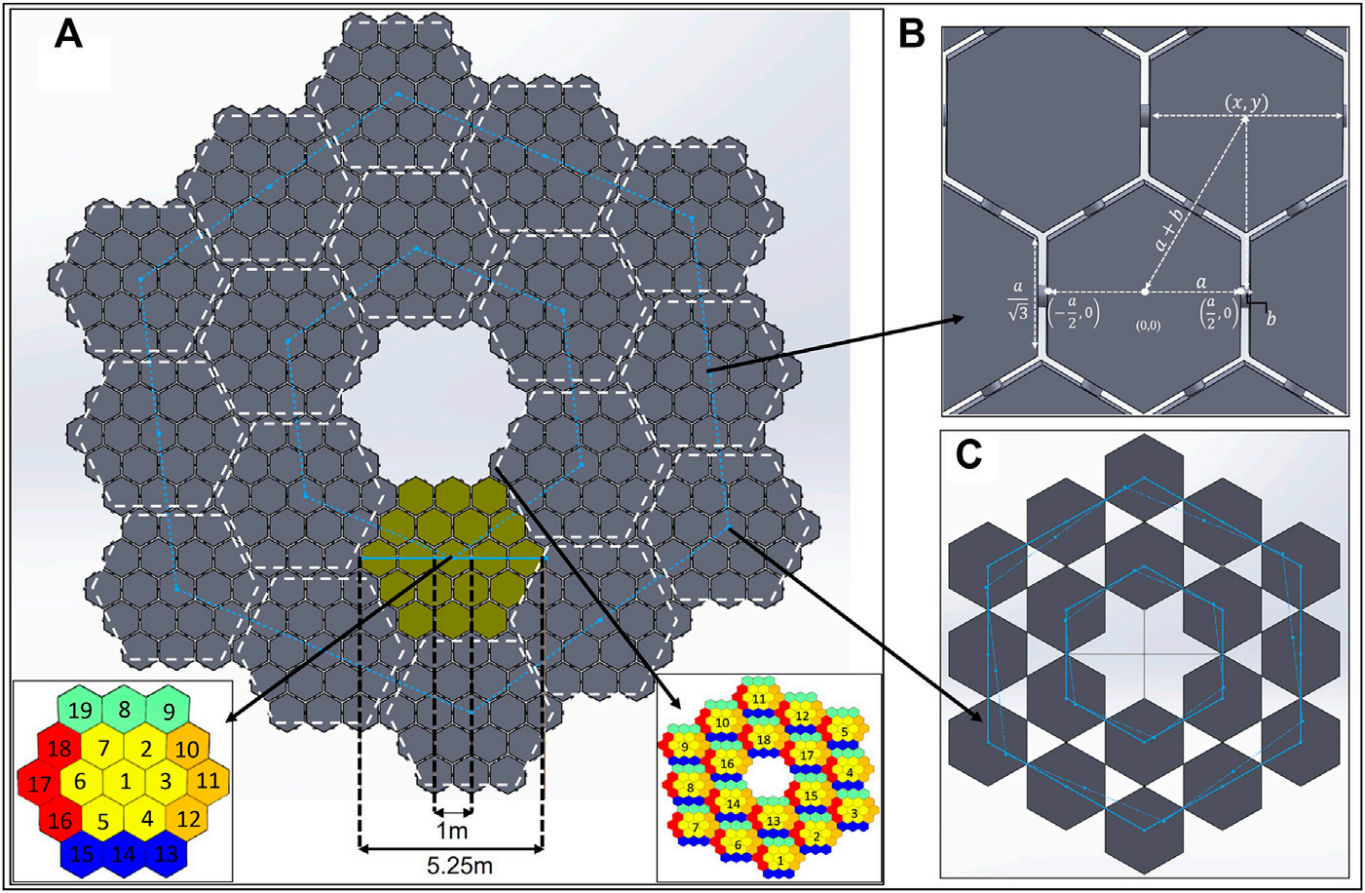
25 m PM and Truss **(A)** PM Design; **(B)** PMU-PMU distance; **(C)** Position of Truss Connectors.

**FIGURE 4:**
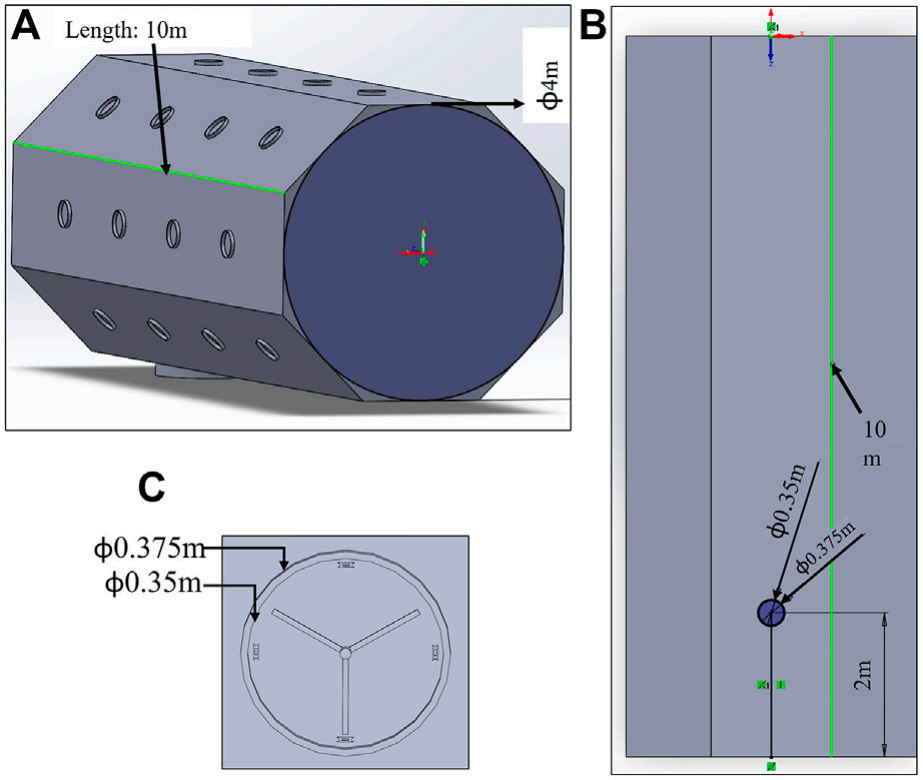
Base spacecraft **(A)** dimensions **(B)** PDGF Configuration–top view **(C)** PDGF dimensions.

Figure 2 illustrates the system architecture of the modular 25m LAST and its spacecraft platform. The modular design of the 25m PM results in a compact stowed configuration that fits well within the fairings of current launchers like Ariane five or 6. Based on the launch-vehicle sizing, the number of Primary Mirror Units (PMUs) for assembly could reach up to 342 units. Each hexagonally shaped PMU ( 1m flat-to-flat) would be equipped with a backplate consisting of connector points on each side. As seen in Figure 2A, 19 PMUs constitute a Primary Mirror Segment (PMS) and 18 of these PMSs form the 25m PM. A deeper insight into the 25m PM assembly in Figure 4 reveals that connectors exist with two different dimension sets. If the PMS in Figure 3A is considered, PMU_1_ is the central mirror to which PMU_2_-PMU_7_ gets connected on one side. Therefore, it can be understood that throughout the assembly of the 25m PM, there would be PMU connectors which receive a connection from other connectors (R_x_ Connector) and which get connected to other connectors (Transmits Connection–T_x_ Connector). In this paper, both the R_x_ and T_x_ connectors are cylindrical, with a height of 50mm . The R_x_ connector has an outer diameter of 110mm and an inner diameter of 100mm . Alternatively, the T_x_ connector has an outer diameter of 100mm (excluding tolerance) and an inner diameter of 90mm (excluding tolerance). Based on the assembly strategy, this unique feature establishes that every PMU would have a unique identity with the different R_x_ and T_x_ combinations. It helps to distinguish each PMU with unique identifiers to assist with the assembly sequencing. In a real-mission assembly, the E-Walker could scan these unique identifiers to ensure that the PMUs are picked up sequentially. Additionally, the connector ports prevent the E-Walker from directly interacting with the mirror itself. The modular design of each PMU facilitates the requirement $R_{2}$ , given that the E-Walker’s motion should be precisely controlled.

Figure 2A shows a close-up view of the PMU connector’s dimensions and design. Based on the assembly sequencing, the E-Walker would be provided with the information on the available connector ports to which it can latch on to. Understandably, the connector ports connected to other PMU ports should be left free. In addition to the R_x_ and T_x_ connectors on the sides of the PMU’s hexagonal backplate, there exists a T_x_ connector on the back side of every PMU_1_ of a PMS (T_xb_ connector), which gets connected to an R_x_ connector of the Truss. As a result, there would be 18 T_xb_’s on the 25m PM as shown in Figure 3A; unlike a regular front-faced hexagon, there exists an alignment shift. Figure 3B shows the parameters used to find the centre of each PMU, represented as ( x,y ), which follows the norm $\left(\left(a/2+b/2\right),\left(\sqrt{3}a/2+b+\sqrt{2ab}\right)\right)$ . Here a is the flat-to-flat distance of the PMU, which is 1m and b is the height of the PMU connector, which is equal to 50mm in this paper). This equation helps identify the 3D position of the Tx_b_ on PMU_1_ of each PMS. The height of the T_xb_ along the *z*-axis is held constant ( ∼=50mm in this paper).

### Spacecraft platform requirements

The spacecraft platform comprises the Truss element, Base Spacecraft (B_sc_) and a Storage Spacecraft (S_sc_). The requirements associated with the platform are elicited below:

#### Truss requirements

The truss is built using 18 modular truss units to hold the assembled Primary Mirror Segments (PMS) by providing a rigid connection using 18 R_x_ connectors. The alignment shift in positioning the Truss R_x_ connectors can be visualised while mapping the T_xb_ onto the Truss. As seen in Figure 3C, the bold line is a regular representation of the R_x_ connectors if placed in each truss element’s centre. However, the dotted line represents the alignment shift while mapping the Tx_b_’s of the space telescope. Based on the ( x,y ) values, the 3D configuration of the T_xb_’s can be identified and, therefore, for the Truss connector ports. In addition to the 18 R_x_ connectors to support the PM, there exist 10 R_x_ connector ports to assist with the E-Walker’s movement. Based on the strategy used for assembly, the E-Walker can also utilise the 18 R_x_ connectors for the PM support either for mobility or pick and place operations.

Establishing the importance of the in-zone mirror positions is necessary to carry out a safe and efficient assembly. These are positions on the truss that can be considered the adjacent mirror positions of any truss connector port. The red and orange circles, in Figure 2B indicate the in-zone mirror positions for the truss connector ports 5 and 8 , respectively. The E-Walker on a truss connector port must not place a PMU or PMS in its in-zone positions, as it would lead to a collision. For example, considering the E-Walker is on truss connector port 5 (C_5_), then mirror positions 3 and 12 are in-zone positions. On the other hand, if the E-Walker is on truss connector port 8 (C_8_), then mirror positions 6 and 8 are considered in-zone mirror positions. Planning the assembly process by avoiding in-zone positions would help the E-Walker satisfy $R_{4}$ .

#### Base spacecraft and storage spacecraft requirements

The adequate number of connector ports on the B_sc_, S_sc_ and truss would help the E-Walker easily access the entire system (Figures 2B,C). Figure 4 shows the dimension of the B_sc_ and its grapple fixtures. As shown in this figure, the state-of-the-art design of current three-pin grapple fixtures on board the ISS to support Canadarm2 and ERA is utilised in this paper. These Power and Data Grapple Fixtures (PDGF) have a flat base plate, a small grapple shaft or pin with a ball on the top and three ramped bars around them to provide additional rotational stability post grapple. In addition, there exist four data and power cable ports and a target pin for the robotic arm to position itself using the camera on-board. To latch on to these fixtures, the Canadarm2 and the ERA have a Latching End-Effector on it is either end. The LEE features a carriage with a rotating ring inside it. The ring incorporates three snare wires, such that when the ring rotates, it closes the snare to lock itself onto the grapple pin, retracting the carriage to latch itself to the spacecraft. Similar PDGF and LEE configurations are designed for the spacecraft platform enabling the E-Walker to move around and perform pick-and-place operations.

The connector ports on top of the B_sc_ are dimensioned with $\phi_{out}=37.5cm$ and $\phi_{in}=35cm$ , to which the E-Walker can get connected for power, data, and mechanical support during the assembly process. These numbers are a rough estimate based on the LEE dimensions of the Canadarm2 and ERA. In order to refrain from having a bulky design of the PMU, the PMU connectors are much smaller than on the spacecraft platform. The LEE of the E-Walker is assumed to have a design that adapts to both these dimensions (Figure 2A). During the mission, the modular mirror units would be stowed inside the S_sc_ in a stacked-up approach. The S_sc_ would then be lifted off to dock onto the B_sc_ during the mission to serve as a collection point for the E-Walker. The B_sc_ ensures attitude stabilisation of the coupled system at all times and helps with the E-Walker’s precise operation during the assembly.

### Other assembly requirements (E-Walker workspace)

**FIGURE 5:**
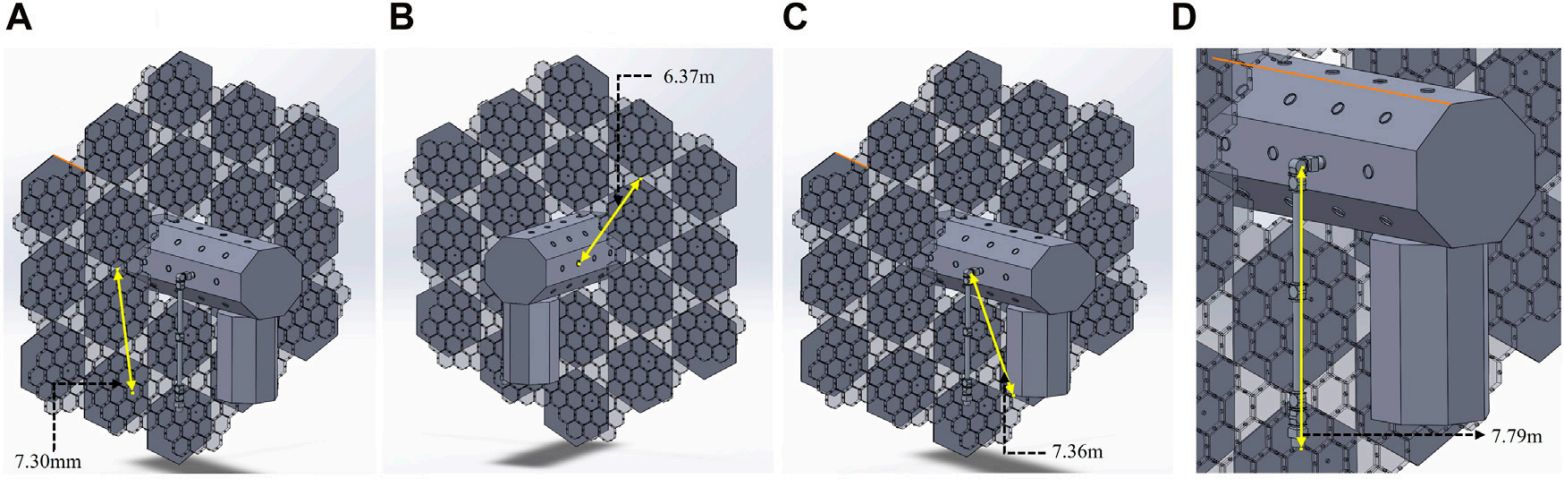
Distance between B_sc_, S_sc_ and Truss Connector ports **(A)** Truss C_8_—PMS_6__PMU_1_; **(B)** B_sc__C_8_—Truss C_3_; **(C)** E-Walker J_2_—S_sc_ lower edge; **(D)** E-Walker J_2_—E-Walker LEE_2_.

For this assembly mission, it is crucial to obtain E-Walker’s maximum workspace, which is necessary to carry out the operations utilising its walking feature. Figure 5 shows measurements which are useful to dimension the E-Walker for the real mission. Based on the dimensions of the spacecraft platform in this paper, Figure 5A shows the distance between C_8_ and the T_x_ connector at the back of PMS 14 , Figure 5B measures the distance between B_sc__C_13_ to Truss connector C_3_ and Figure 5C shows the distance between B_sc__C_9_ and the bottom of the S_sc_. The length of the E-Walker from J_2_ to LEE_2_ is shown in Figure 5D. These measurements reveal that the E-Walker’s workspace is within 7.5m . Therefore, it is concluded that an E-Walker with a total length of 8m would suffice to assemble the 25m PM. Compared to a fixed-to-base robotic manipulator, which would otherwise require a 12.5m long arm to assemble the PM, there’s a reduction of 4.5m in the total length for the E-Walker. This design optimisation minimises the mass, volume, launch cost, and power requirements for the whole mission.

## End-over-end walking robot

In this paper, the robotic architecture presented to facilitate the autonomous assembly of the 25m LAST in orbit is an End-Over-End Walking Robot (E-Walker). The choice is conclusive evidence of the extensive review by (Nanjangud et al., 2019a) and the proposed architecture in (Brooks et al., 2016; Nanjangud et al., 2019b; Letier et al., 2019; Rognant et al., 2019). The E-Walker’s space design is symmetric and draws inspiration from the Candarm2 and the ERA onboard the ISS and the robotic manipulator presented in the Modular and Re-Configurable Spacecraft **(**MOSAR) project (Cruijssen et al., 2014; Glen Gook, 2015; Deremetz et al., 2021). The symmetric feature was also introduced in a five DoF earth-based walking robot manipulator—the Climbot (Guan et al., 2011). This section compares the E-Walker design with the other identified walking robotic manipulators and identifies the key differences to propose a novel robotic solution for the upcoming in orbit missions.

A top-level comparison between the state-of-the-art walking robot manipulators in space and the E-Walker space design is shown in Table 2. In terms of design comparison, the Canadarm2 has an offset around the elbow joint, which provides it with the capability to walk end-over-end, however, the connector ports have to be placed in a spacing equal to the joint offset to enable a straight-line motion. In comparison, the ERA and MOSAR have in-line elbow joint without an offset. However, as the two links are clamped onto the elbow joint on the sides, both the arms are limited of a full rotation around the elbow and end up in end-to-end walking. This results in an increased dependency on the roll joints of these robot manipulators, which is not recommended for a space mission. The E-Walker is an amalgamation of all the key features of the above-mentioned robotic arms, i.e., achieving end-over-end walking with an in-line elbow joint to achieve easy motion along a straight line without joint offset. For the earth-based design, the Climbot is a walking robot manipulator which has a similar elbow joint feature as the E-Walker. However, its workspace, redundancy and dexterity are limited due to the reduced number of joints (5 DoF).

**TABLE 2:** Space-system comparison.

Features	Canadarm2	ERA	MOSAR	E-Walker
Links	2	2	4	2
Joints	7	7	7	7
End-Effectors	2	2	2	2
Total Length ( m )	17	11.3	1.6	∼8 (customisable)
Weight ( kgs )	1497	630	30	∼475
Payload ( kgs )	116000	8000	10	>1000 (to be tested)

In comparison, the E-Walker prototype is dexterous with seven DoF and facilitates a modular design with customisable end-effectors. A comparison between Climbot and the E-Walker prototype design is enlisted in Table 3.

**TABLE 3:** Earth-analogue comparison.

Features	Climbot	E-Walker
Links	4	2
Joints	5	7 (customisable)
End-Effectors	2	2
Total Length ( m )	1.55	Prototype length - ∼1.3 (customisable)
Weight ( kgs )	17.5	∼12
Payload ( kgs )	Unknown	2 (1st Prototype), customisable
End-Effector type	Fingered Gripper	Magnetic (1st Prototype), customisable

The E-Walker’s capability to connect to static connector ports onboard the B_sc_ and Truss helps to cover a workspace much larger than its span. This enhanced reachability is an added benefit for assembly missions. In this paper, the E-Walker is assigned two key operations throughout the mission. These operations involve assembling PMSs using PMUs, thereafter, performing pick-and-place operations of the assembled PMSs onto their respective truss locations. The E-Walker would facilitate mobility around an attitude-stabilised B_sc_, with a predefined motion that will not alter the attitude of the B_sc._ This is contrary to the state-of-the-art concept of a space robot which requires additional effort in the Guidance, Navigation and Control unit. This is due to the complexities involved in maintaining attitude due to the non-linear dynamic coupling effects between the spacecraft platform and the fixed-to-base robotic manipulator (Flores-Abad et al., 2014).

The seven DoF E-Walker has three revolute joints, each in the shoulder and the wrist, and one DoF in the elbow joint. The dexterous symmetric design provides access to any part of the workspace (see Figure 1), satisfying the requirement $R_{3}$ completely. The seven DoF E-Walker can use either of its two LEEs to latch on to the B_sc_. During each phase, the LEE on either side of the E-Walker, attached to J_1_ or J_7_, is locked to the spacecraft platform. A fixed-to-base robot manipulator would require a full-length span of ∼12m to assist with the assembly when placed at the centre of the 25m LAST system (Nanjangud et al., 2019a). On the contrary, the E-Walker’s dimensions can be reduced subject to the availability of the connector points and the payload sizing.

The initial design of the E-Walker considered a five DoF model, which included two revolute joints, each in the shoulder and wrist, one revolute joint in the elbow and two LEE on either end (Nair et al., 2020a). The five DoF E-Walker provided an insight into the minimum DoF required to realise the cyclic motion dynamics; this was a baseline design to carry out an end-over-end motion. This design met the requirement $R_{4}$ partly. However, for space-based operations, both redundancy and dexterity are essential (Dalla and Pathak 2019). Redundancy offers enhanced reachability and acts as a significant backup in case of a joint failure. Therefore, to overcome the challenges of the five DoF E-Walker’s limitations, the seven DoF E-Walker design was introduced as an upgrade. The enhanced mobility feature of the seven DoF E-Walker can also help satisfy the requirement $R_{5}$ post the assembly.

### Link design optimisation

Designing the E-Walker link requires optimising the link shape and dimensions prior to carrying out the SSA. An optimum link design should have the lowest mass and undergo minimal deflections under loading.

#### Link configuration

In this study, a solid tube, a hollow tube, and a rectangular beam were considered for the shape of the link (refer: Table 4). For tube shaped links, r is the radius, ϕ is the diameter, whereas h is the link height. The rectangular beam has l and b as the link length and breadth. These shapes were then analysed based on their mass and area moment of inertia (I_A_). These parameters determine the deflection under loading, which is independent of the link length. The equations for I_A_ for the shapes considered are shown in Table 4.

**TABLE 4:** Link shape, I_A_ and volume equations.

Link Shape	I_A_	Volume
Solid Tube	$\pi r^{4}/2$	$\pi r^{2}h$
Hollow Tube	$\pi {(r}_{1}^{4}-r_{2}^{4})/2$	$\pi {(r}_{1}^{2}-r_{2}^{2})h$
Rectangular Beam	$bh^{3}/12$	lbh

Table 5 presents the dimensions of the three shapes to down select a single link based on their calculated parameters. To down select a single link shape. The space-qualified material, Aluminium 7075, with a density of $2750kg/m^{3}$ is considered for the fabrication and it meets the requirement $R_{5}$ (Younger and Eckelmeyer 2007; Dursun and Soutis 2014). The link length is considered to be 3.5m in all the cases ( $R_{3}$ ). These values were then used to determine the ratio of mass to area moment of inertia for each of the different shapes. A smaller ratio is optimum since the corresponding shape will exhibit lower deflection levels for the same mass. A ratio was used as it removes dimensions from the analysis. Therefore, the conclusions from Table 5 are valid regardless of the final selected link parameters. Table 5 shows that the hollow tube link exhibits the lowest mass to I_A_ ratio and is selected as the optimum link configuration for the E-Walker.

**TABLE 5:** Link parameters.

Shape	Dimensions ( cm )	Volume	Mass (kg)	Area Moment of Inertia	Mass to Area Moment of Inertia (10^5^)
Hollow Tube	$\phi_{out}=30$ t=1.5	0.0615	169.33	$3.46\times 10^{-4}$	4.89
Solid Tube	ϕ=30	0.247	680.35	$7.95\times 10^{-4}$	8.56
Rectangle	b=30h=20	0.21	577.5	$2\times 10^{-4}$	28.87

#### Link dimensions

*Link configuration* section presented the validation of selecting a hollow aluminium tube as the link shape. Therefore, to select the appropriate sizing of the link, different dimensions of a hollow tube were taken into consideration to conduct Finite Element structural analysis in Ansys. Table 6 shows four main sets (Set 1—Set 4) of link sizes considered for the analyses. Dimensions smaller than 20 cm were not considered as the links would have wirings running through their inner walls to support the actuators and other related electronics.

**TABLE 6:** E-Walker link dimension sets.

Set	$\phi_{out}\;\left(cm\right)$		$\phi_{in}\;(cm$ )
Set 1	50		47
Set 2	40		37
Set 3	30		27
Set 3.1	$\phi_{out1}=30$	$\phi_{out2}=25$	23
Set 3.2	$\phi_{out1}=30$	$\phi_{out2}=20$	18
Set 4	20		17

#### Link static structural analysis

The maximum deflection and stress on the different link sizes can be analysed by computing the deflection of a single link. If the E-Walker is assumed to be erect with LEE_1_ being fixed to the base, the maximum loading would be experienced by L_1_ due to J_4_-J_7_, L_2_, LEE_2_ and the payload. If LEE_2_ is fixed to the spacecraft platform, L_2_ would experience a similar load (J_1_-J_4_, L_1_, LEE_1_ and payload) due to the symmetry of the E-Walker. Therefore, if the design is validated for L_1_, then those results would be sufficient for L_2_. To carry out the link deflection tests, the E-Walker is assumed to be in the initial state of Phase 1, i.e., standing straight, carrying a payload of a PMS ( 342kg ∼=3420N ). Therefore, the mass of J_4_-J_7_, Link 2, the LEE, and the payload act on L_1_. A torque of 100Nm is also provided, considering a situation where J_2_ is under motion, trying to bring the whole E-Walker to complete Phase 1, thereby imparting a twist on L_1_. This is a good estimate for the maximum load on the L_1_. The deflection and stresses on L_1_ due to this loading and torque was further analysed using FEA simulation. The simulation was set up by providing a 5c m tetrahedral mesh to L_1_, fixing one end and applying the respective force and torque values on the other end. The selection of this mesh type is attributed to its simple 3d mesh, which can be easily applied to any shape and allows for quick and accurate modelling (Hughes, Cottrell, and Bazilevs 2005). To verify the selection of the mesh size, the mesh convergence on the area under maximum stress in the whole link, i.e., the top end of the link, was considered. For reference, the design goal for this study, i.e., the maximum deflection of L_1_, under maximum loading is limited to 0.05 mm.

**FIGURE 6:**
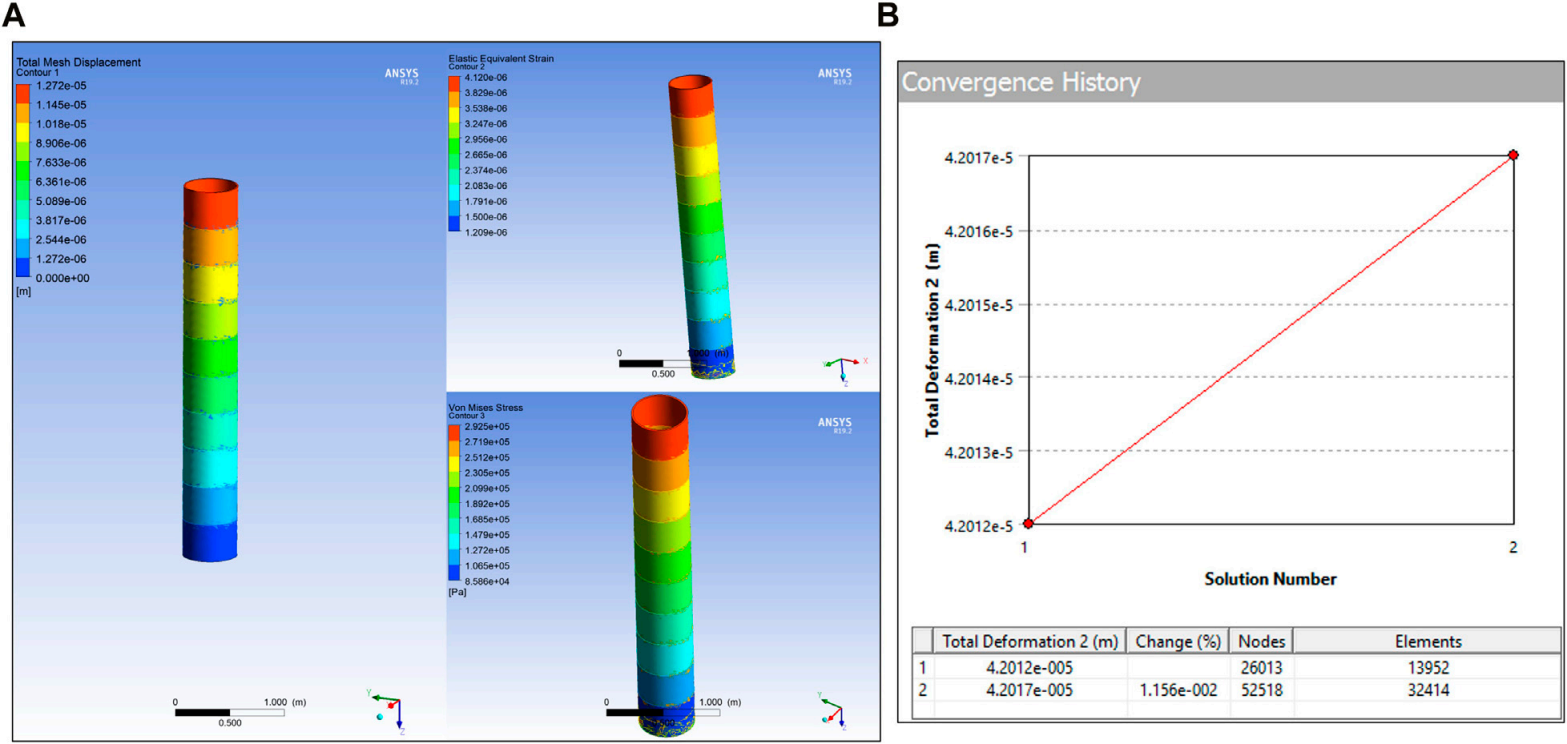
Link Set 1 **(A)** SSA; **(B)** Mesh convergence.

**FIGURE 7:**
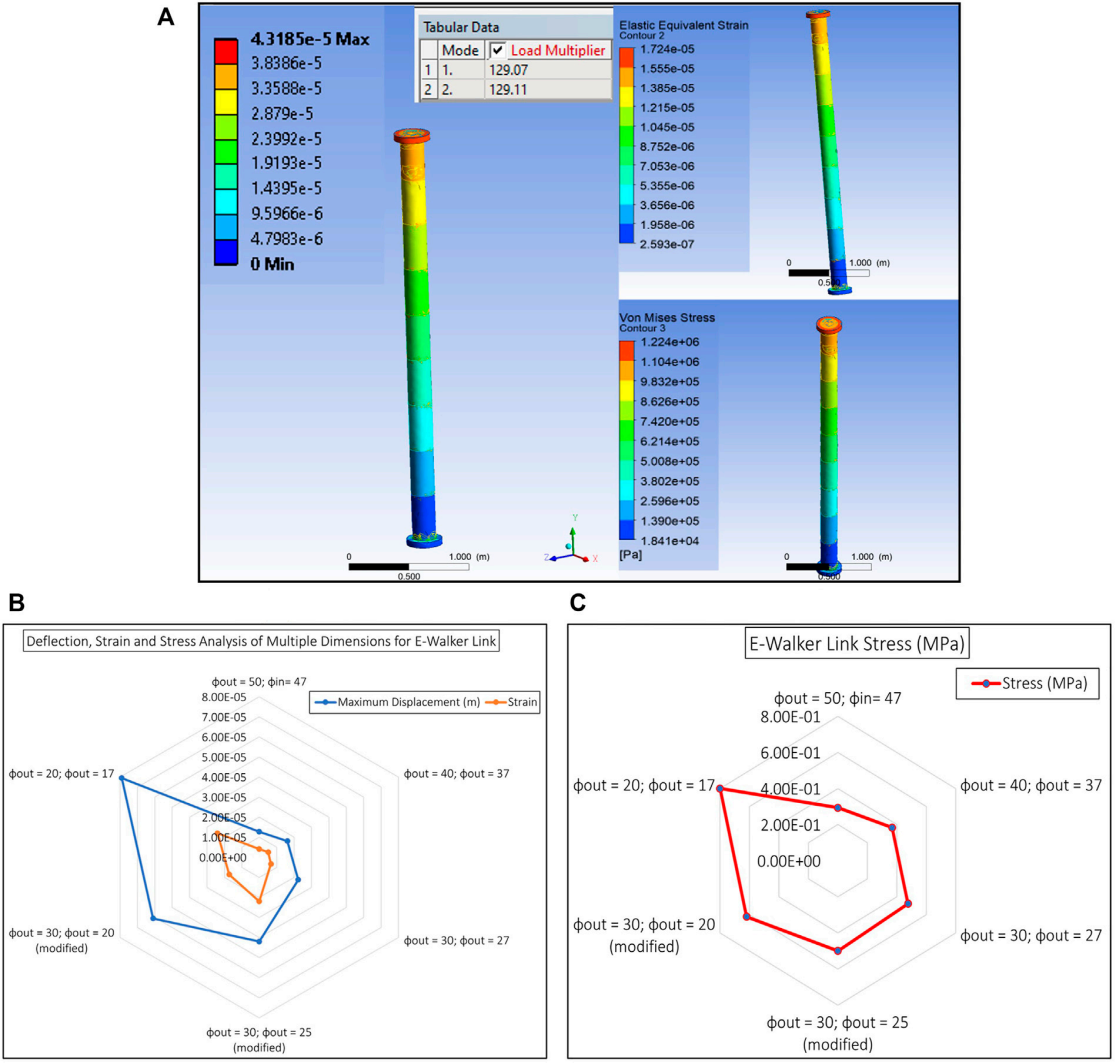
Link Set: **(A)** Ansys FEA of modified link; **(B)** Displacement and Strain Radar Chart of Link sets; **(C)** Stress Radar Chart of Link sets.

Modelling the Set 1 link produced minor deflection of 0.01mm . The total mesh displacement equivalent elastic strain and the Von Mises stress plots are shown in Figure 6. The convergence percentage of the mesh at the top of the link was set as low as 5% to verify the mesh sizing of the link. As seen in Figure 6, the mesh size is well converged, validating the link design. Similar steps were followed to measure the above parameters for the rest of the link sets and the results obtained are illustrated in Figure 7. From Figures 7A,B, it is observed that the Set four link produced a deflection of around 0.08mm , which exceeds the design goal limitation, and is not further considered for analysis. In contrast, the Set three link produced a deflection of around 0.022mm and provided a mass reduction of around 92kg when compared to Set 1 design. Therefore, the Set three link was modified to further reduce the mass. The design and FEA simulation results of the modified link is shown in Figures 7C,D respectively. The dimensions of Set three are a near estimate of Canadarm2 and the ERA.

As seen from Figure 7C, a design with wider ends of 30cm diameter was proposed as they can withstand higher stress levels. The inner rod has a slender design with two size sets, Set 3.1 and 3.2, respectively. In order to validate these changes, the same FEA analysis was carried out to ensure that the modifications do not induce unacceptable amounts of deflection or stress. Set 3.1 had a maximum mesh displacement of 0.04mm , which was well within the design goal, whereas Set 3.2 produced changes of about 0.062mm , which exceeded the design goal; Set 3.2 was not considered for further evaluation. The Eigen Value Buckling of Set 3.1 link showcased a load multiplication of 129 (refer Figure 7D), which is a reliable safety factor for the E-Walker link to perform the assembly. To conclude, the final link design chosen for fabrication is Set 3.1.

### Latching end effector design analysis

**FIGURE 8:**
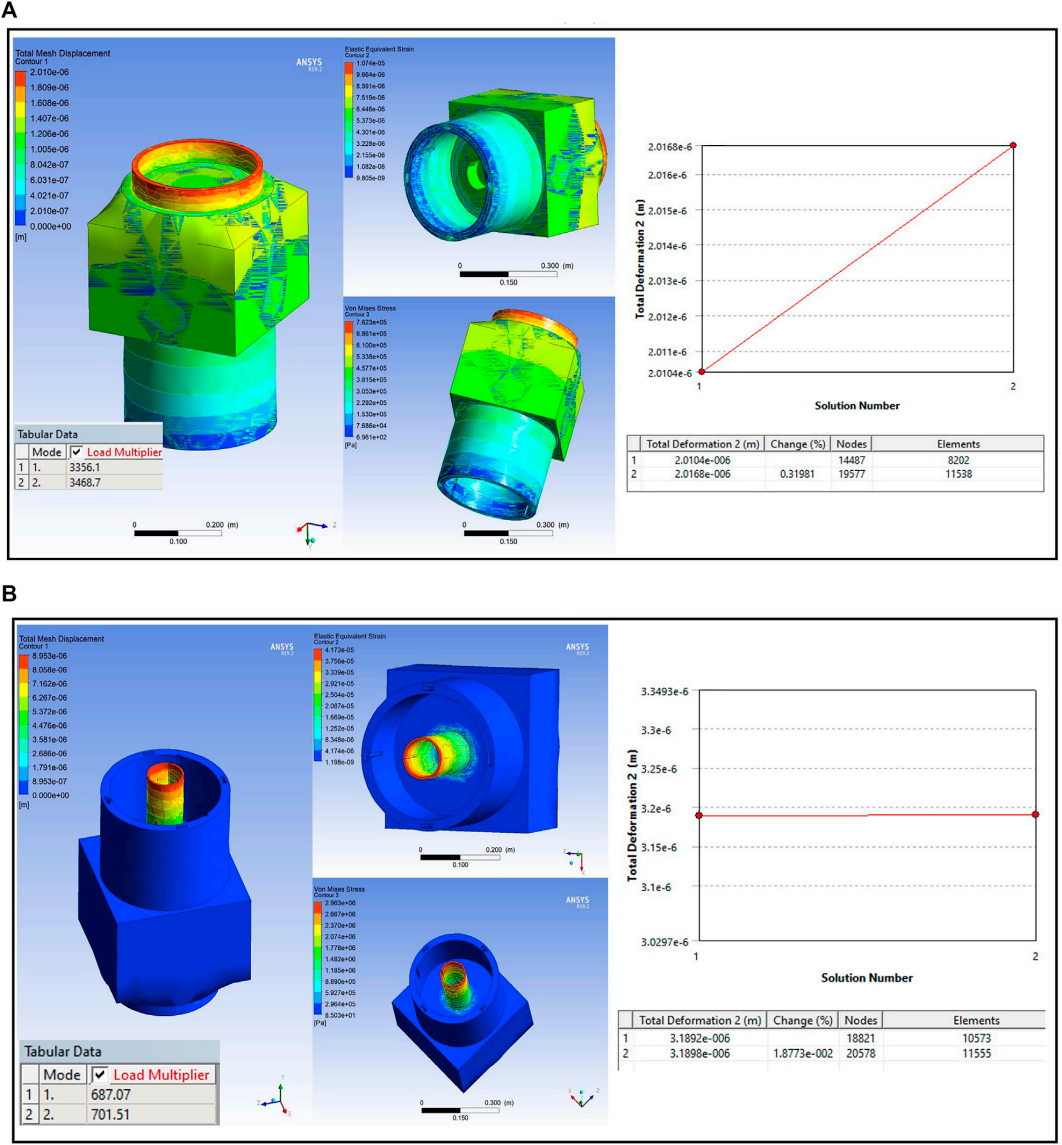
Latching end effector **(A)** large ring SSA; **(B)** small ring SSA.

The E-Walker consists of a Latching End-Effector (LEE) on either end to provide the required connection to the spacecraft platform and PMU connectors. The LEE is therefore subjected to loading conditions and requires further analysis to optimise the design (Figure 8). The LEE design in this paper has two ring features, one to latch onto the spacecraft platform ( $\phi_{out}=35cm$ , thickness =2.5cm ) and a dimensionally smaller inner ring ( $\phi_{out}=10cm$ , thickness =0.5cm ) to latch onto the PMUs (refer Figure 8A). It is important to independently estimate the structural analysis of both the ring features.

For the SSA, one LEE of the E-Walker is assumed to be fixed on to the spacecraft and the other end is to be carrying the maximum payload of one PMS. For the larger ring, the maximum normal force would be applied when it bears the mass of the whole E-Walker carrying one PMS. The smaller ring experiences the maximum normal force due to the payload alone. Structural analysis of one LEE is enough due to the symmetry of the E-Walker. Referring to E-Walker’s initial position in Phase-1, a force of 8000N is applied vertically on the larger ring and that of 3500N is applied on the smaller ring. Figure 8B shows the structural analysis of the LEE’s larger ring fixture with displacements as low as 0.002mm . The Eigenvalue buckling results show a load multiplier of at least 3300 , which is a significant safety factor for the E-Walker. The smaller ring had a maximum deformation of around 0.008mm and a buckling multiplier of at least 687 , which is a significant safety factor (Figure 8C), validating the design of the LEE.

### Actuator selection

Selecting actuators for space missions revolves around understanding the dynamics of the system. The dynamical model of the E-Walker helps evaluate individual joint torque limits and the type of actuator needed for each joint.

#### Dynamic modelling of E-walker

Using the Euler-Lagrange equation (Spong, Hutchinson, and Vidyasagar 2006), the non-linear dynamic equation of the seven DoF E-Walker is given as:

$$D\left(\theta \right)\ddot{\theta }+C\left(\theta ,\dot{\theta }\right)\dot{\theta }+G\left(\theta \right)=\tau$$ (1)

where, $\tau \in R^{n}$ is the joint torques, $\theta \in R^{n}$ represents the joint angles and $\dot{\theta }\in R^{n}$ represents the corresponding joint velocity vector. Considering a link i with mass $m_{i}$ and an inertia matrix calculated about its centre of mass (CoM), $D\left(\theta \right)\in R^{n\times n}$ is the mass matrix, which is symmetric and positive definite for all joint angles $\theta \in R^{n}$ . It is given as:

$$D\left(\theta \right)=\overset{n}{\underset{i=1}{\sum }}\left[m_{i}J_{v_{mi}}{\left(\theta \right)}^{T}J_{v_{mi}}\left(\theta \right)+J_{\omega_{mi}}{\left(\theta \right)}^{T}R_{i}\left(\theta \right)I_{i}R_{i}{\left(\theta \right)}^{T}J_{\omega_{mi}}\left(\theta \right)\right]$$ (2)

Here, the Jacobian matrix $J\in R^{6\times n}$ matrix is comprised of the linear Jacobian, $J_{v_{mi}}\in R^{3\times n}$ and the angular Jacobian, $J_{\omega_{mi}}\in R^{3\times n}$ . The linear velocity of the *i*th joint is given by $v_{i}=J_{v_{mi}}\;\dot{\theta }$ and the angular velocity for the *i*th joint is expressed as $\omega_{i}=J_{\omega_{mi}}\;\dot{\theta }$ . The mass matrix is a crucial component in the robot dynamic equation and plays a significant role in robot control.

$C\left(\theta ,\dot{\theta }\right)\in R^{n\times n}$ , comprises of the Coriolis and Centrifugal forces that act contrary to the motor commands and can be given by:

$$c_{ij}=\sum_{i=1}^{n}c_{ijk}\left(\theta \right){\dot{\theta }}_{k}=\sum_{i=1}^{n}\left(1/2\right)\left\{\left(\partial d_{ij}/\partial \theta_{k}\right)+\left(\partial d_{ik}/\partial \theta_{j}\right)-\left(\partial d_{kj}/\partial \theta_{i}\right)\right\}{\dot{\theta }}_{k}$$ (3)

During a space mission, due to the microgravity conditions, the effect of potential energy is usually neglected. However, near-less gravity plays an essential role in the control of robot dynamics in orbit and is taken into consideration in this paper. In Eq. 1, the gravity matrix $G\left(\theta \right)\in R^{n}$ considers a microgravity value of $10^{-6}g\;m/s^{2}$ , where $g=9.81m/s^{2}$ , is the acceleration due to gravity on Earth. Based on the masses obtained in *Latching end effector design analysis* and *Actuator selection*, the link and LEE mass were increased to incorporate the mass of additional electronics, wiring *etc.* The ERA, with an 11.3 m length weighs around 630 kgs. Considering the ERA as a reference design, the total mass of the E-Walker that uses similar components is around 475 kg.

**FIGURE 9:**
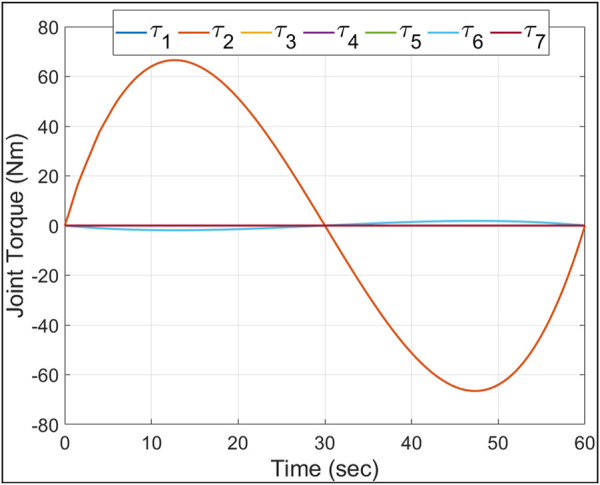
Joint two maximum torque.

The maximum torque required by an E-Walker’s actuator can be computed with E-Walker completely stretched out by J_2_ and carrying a payload using a LEE. In this configuration, using Eq. 1 and considering E-Walker’s inertial parameters, the simulation was carried out with the E-Walker in its initial configuration of Phase 1, moving to the extreme position in 60s . The maximum torque experienced at J_2_ was around 70Nm (refer Figure 9), which is well within the torque ( 100Nm ) applied during the structural analysis of L_1_ in *Link static structural analysis* section. Using this value as a reference, space-qualified actuators could use a frameless motor with a harmonic drive attached to it (Space Actuators 2017; Harmonic Drive SE 2022). The dynamic torque analysis helps satisfy the requirement $R_{S6}$ .

### E-walker modal analysis

**FIGURE 10:**
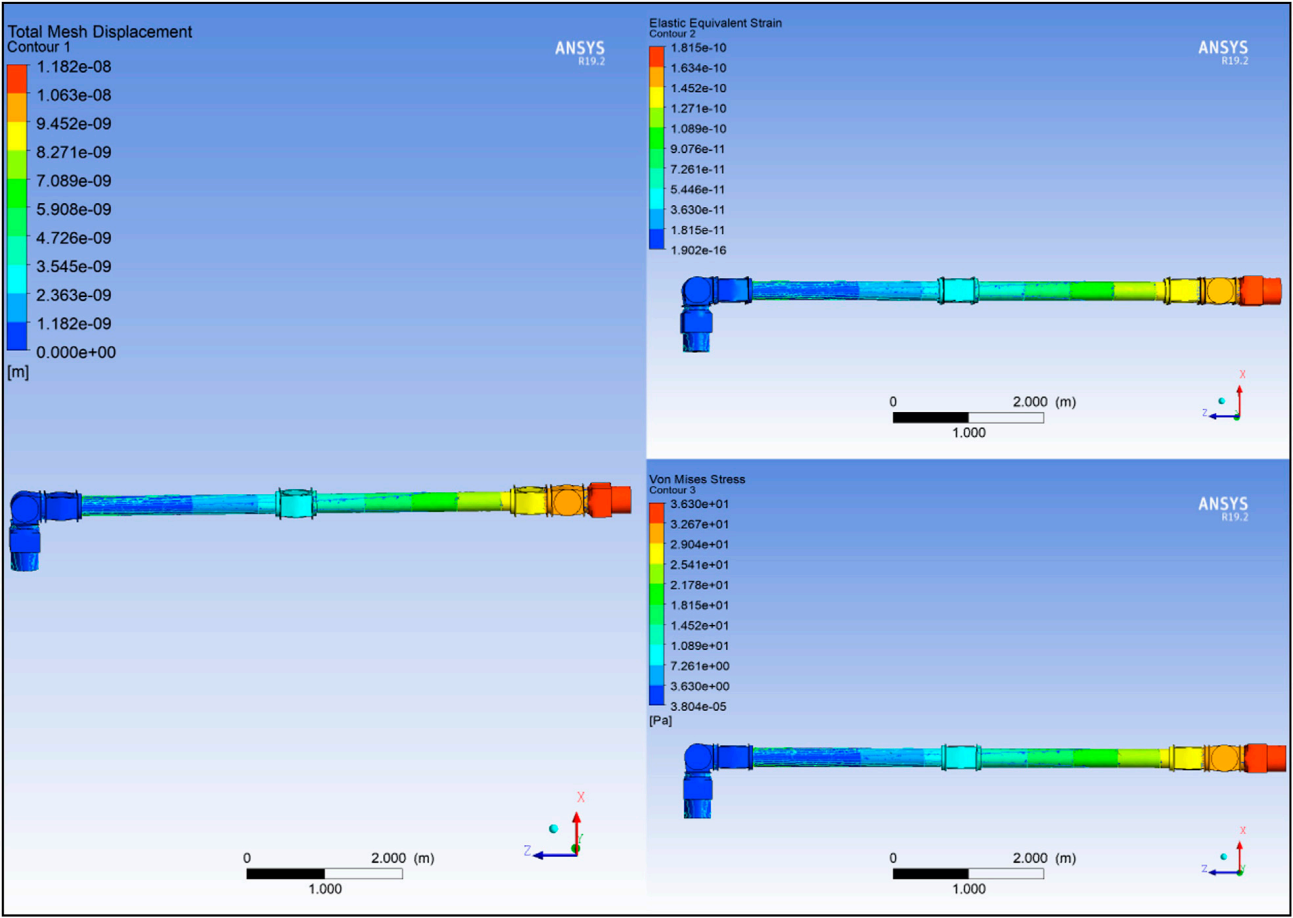
E-Walker SSA with no Payload.

**FIGURE 11:**
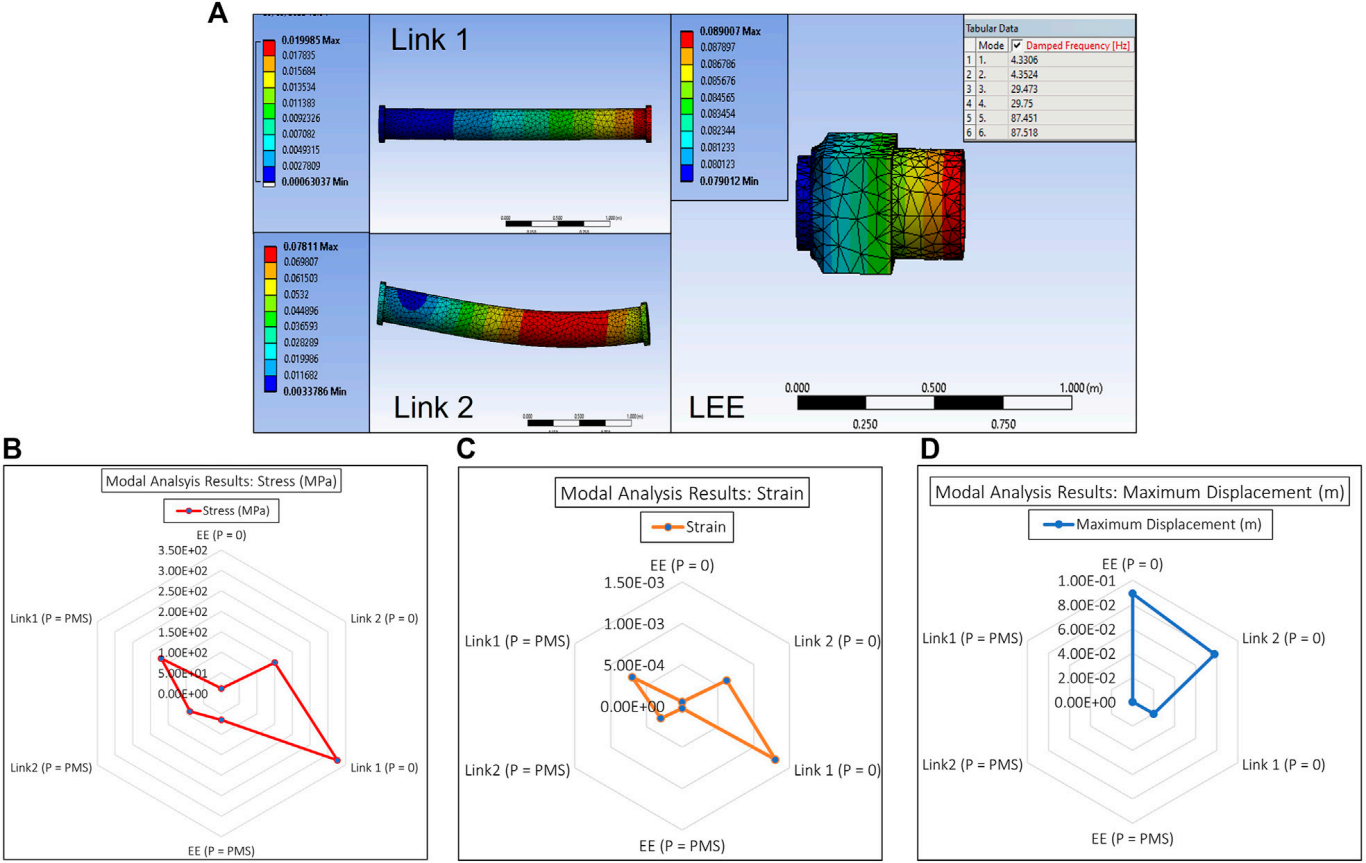
E-Walker Modal Analysis **(A)** Ansys Simulation of Links and LEE **(B)** Deflection analysis **(C)** Stress Analysis **(D)** Strain Analysis.

Based on the E-Walker’s design optimisation in *Link design optimisation*, *Latching end effector design analysis*, and *Actuator selection* sections, the entire model was put under open-loop testing and no-load conditions in an ideal environment. As seen in Figure 10, the deflection is minimal. However, the challenge for the E-Walker is more severe as it has to move a PMS under the extreme environment in space and therefore requires frequency or modal analysis. The modal analysis in Ansys helps estimate the dynamic behaviour of the E-Walker when prone to different frequencies in the extremities of the space environment. The worst case performance of the E-Walker was evaluated in its most vulnerable position, i.e., with the arm completely stretched out about J_2_ with a maximum payload ( ∼=1 PMS) at its end. Six modes were considered, and the total displacement of the links and the LEE were analysed under each mode with a different frequency (Figure 11). As observed in Figure 11A, link two experiences a maximum link deflection of 7.8cm and the LEE experiences a deflection of around 8.9cm . It is to be noted that the modal analysis provides significant insight into the dynamical characteristics of the E-Walker in an open loop. The deflections obtained through this analysis could be modelled as disturbances while developing and optimising a suitable closed-loop robust controller for the E-Walker.

## E-walker scaled-down Earth analogue design

Verifying the large-scale model of the E-Walker is near to impossible under Earth analogue conditions, given the high torque demands due to Earth’s gravitational pull and the limited microgravity environment test facility on the ground. Due to these reasons, a scaled-down prototype of the E-Walker is considered for fabrication to verify and validate tasks involving mobility and pick-and-place operations. The design engineering exercise similar to the one carried out in *End-over-end walking robot* is repeated for the prototype in Sections 4.1–4.4. The comparison of the full-scale E-Walker and its scaled-down Earth-analogue design helps understand the issues with scalability and the impact of Earth’s gravitational acceleration. Table 7 presents a summary of the design trade-offs of the full-scale E-Walker and its prototype. Detailed SSA and actuator selection methods are presented in the following subsections. Similar to the full-scale space design, it is significant to identify the design requirements specific to the E-Walker’s scaled-down prototype ( $R_{Pi}$ ) to carry out operations under Earth’s gravity. Table 8 presents the list of requirements.

**TABLE 7:** E-walker full scale and prototype comparison.

Parameters	Full-scale		Prototype
Scale	1:1		1:6
Total Length	8m		1.3m
Mass	∼475kg		∼12kg
Payload	350kg (maximum for a mission)		2kg
Link Maximum Deflection	0.04mm		0.14mm
Link Load Multiplier	129		75
LEE/Base	LEE Large Ring	LEE Small Ring	Base
LEE/Base Max Deflection	0.002mm	0.008mm	0.002mm
LEE/Base Load Multiplier	3350	680	4030
Actuator Torque	70Nm		42Nm

**TABLE 8:** E-walker prototype requirement list.

Sl. No	Requirement
$R_{P1}$	The E-Walker’s earth analogue design should be a scaled-down prototype the meets industry standards but for space qualification
$R_{P2}$	The E-Walker should have 7 DoF for full dexterity
$R_{P3}$	Payload capacity is 2 kg
$R_{P4}$	Preferably Pancake BLDC Actuators are to be used for implementing high torques
$R_{P5}$	Aluminium or Carbon fibre 3D printing can be considered for prototyping purposes
$R_{P6}$	The base plate should incorporate ON-OFF electromagnets for E-Walker to attach itself to metal rails

### Bending moment, stall torque and actuator selection

**FIGURE 12:**
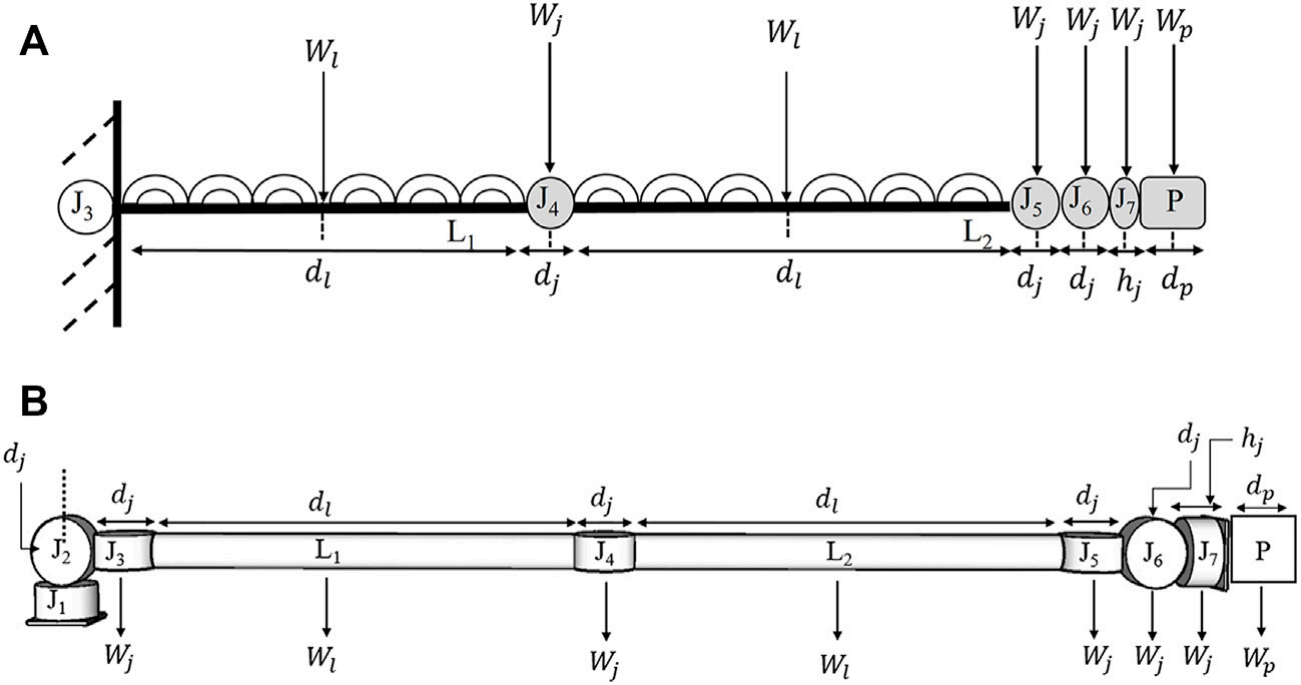
E-Walker Configuration **(A)** To calculate Bending Moment on L_1_ **(B)** To calculate Stall Torque on J_2_.

A similar FEA-based analysis used in *End-over-end walking robot* design can be used to optimise the link design for the scaled-down prototype. Figure 12 helps identify a general equation to calculate the bending moment and the corresponding torque needed to traverse a desired trajectory. Considering the actuators and payload of a given dimension, Figure 12A shows the Bending Moment (BM) for the links of the seven DoF E-Walker. Corresponding to this worst-case configuration, L_1_ will experience a larger bending moment than L_2_. The symmetrical design of the E-Walker suggests that evaluating the bending moment on L_1_ alone at the stretched condition is sufficient to get a conclusive estimate of the maximum twist experienced.

**FIGURE 13:**
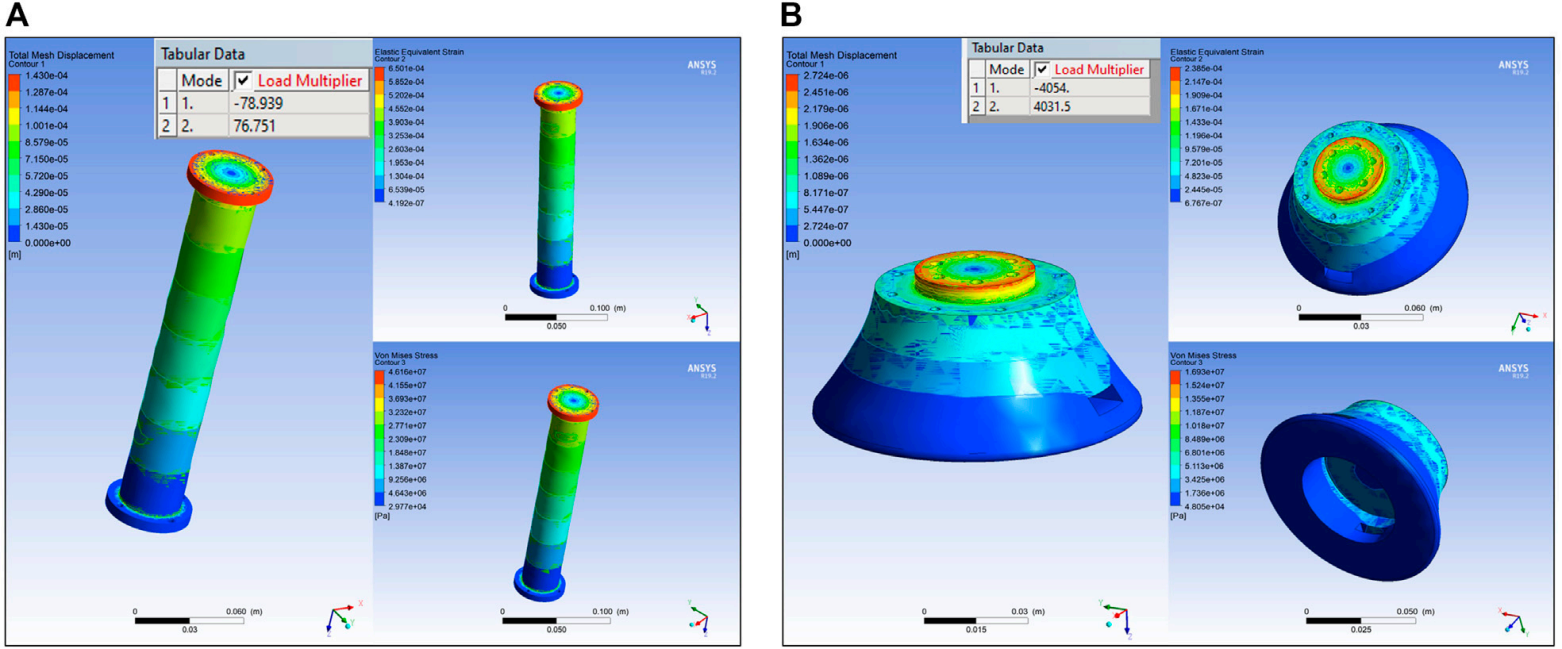
E-Walker prototype ansys FEA **(A)** Link **(B)** Base plate.

The links, actuators and payload are considered to have uniformly distributed load, with its weight considered to be concentrated at its centre of mass. From Figure 13, the following parameters are used to evaluate the BM : P–Payload; L_i_–Link; J_i_–Actuator; $m_{p}$ —Payload mass = 2kg; $m_{j}$ —Actuator mass; $m_{l}$ —Link mass; $W_{p}$ —Payload Weight *=* 20N ; $W_{j}$ —Actuator Weight; $W_{l}$ —Link Weight; $d_{p}$ —Payload length; $d_{j}$ —Actuator length/diameter; $h_{j}$ —Actuator height; $d_{l}$ —Link length. The Bending moment equation is derived as:

$$BM=\left[\begin{array}{ccc} W_{l} & W_{p} & W_{j} \end{array}\right]\left[\begin{array}{c} {2d}_{l}+d_{j}\\ {2d}_{l}+{0.5d}_{p}+{3d}_{j}+h_{j}\\ {7d}_{l}+{7.5d}_{j}+{0.5h}_{j} \end{array}\right].$$ (4)

The maximum stall torque is estimated considering that all the actuators are of the same specification, and the E-Walker has to implement motion using either of its ends whilst carrying a 2kg payload. The E-Walker experiences the maximum stall torque when it carries the 2kg payload with its arm stretched out (Figure 12B). This is much similar to the fully stretched configuration of the human arm, 90° with respect to the shoulder when carrying a load. Therefore, in the case of the E-Walker, if J_2_ is stretched out as shown in Figure 12, it will experience the maximum stall torque, and the rest of the actuators could be selected based on this calculation. The same can be considered with the case of J_6_ due to the symmetry of the design. The stall torque equation is derived as:

$$ST=\left[\begin{array}{ccc} W_{l} & W_{p} & W_{j} \end{array}\right]\left[\begin{array}{c} {2d}_{l}+{4d}_{j}\\ {2d}_{l}+{0.5d}_{p}+{4.5d}_{j}+h_{j}\\ {7d}_{l}+{14.5d}_{j}+{0.5h}_{j} \end{array}\right]$$ (5)

Based on Eq. 4 and Eq. 5, two case studies were carried out for different actuators available commercially (R100 KV90 2022; RMD-X8 Pro V2 2020; AK80-80 2022). In this research, a bespoke design of an using industrially available motors, gears, and encoders were not considered, given the compatibility issues, assembly costs and lead time. The RMD and AK series actuators had a diameter range between 5 and 10 cms. Therefore, a 5 cm and a 10 cm actuator is used to evaluate the range of BM and ST to carry a 2 kg payload ( $W_{p}=20N$ ). Aluminium 7075 is considered for fabricating the 20 cm links. Based on these parameters, the BM and ST corresponding to these design parameters are shown in Table 9. For the E-Walker assembly, the actuator casings would support clamps which would help with joint-joint and joint-link rigid assembly. The data from the case studies in Table 9 reveal that for the E-Walker, an actuator with a 5cm diameter would need a stall torque of 20Nm and that of a 10cm diameter would need an ST of 43Nm . Based on this analysis, the AK80-80 actuator from T-Motors is selected for the prototype (AK80-80 2022). It has an external diameter of 98mm and a mass of 790g . It has a continuous stall torque of 48Nm and a peak torque of 144Nm , which falls within the required specification of the E-Walker.

**TABLE 9:** Case Studies for E-Walker prototype design.

Case	$d_{j}$ ( m )	$h_{j}$ ( m )	$W_{j}$ ( N )	$d_{l}$ ( m )	$W_{l}$ ( N )	$d_{p}$ ( m )	BM on L_1_ ( Nm )	ST on J_2_ ( Nm )
Case 1	0.05	0.025	4	0.2	1.49	0.05	19.82	22.94
Case 2	0.1	0.065	8	0.2	1.49	0.05	34.01	43.05

### Link design optimisation and static structural analysis

Similar to the link design optimisation in *End-over-end walking robot* design, Table 10 shows the dimension sets considered for SSA. An optimal design reference was set for the links not to bend more than 0.5mm with sufficient buckling tolerance. SSA was performed on Sets 1 - 5 with an estimate of the BM in each case along with the weight of the other motors, L_2_ and the payload. The total deformation and stress were evaluated in each case. As per the results presented in Table 10, Set 5 has a deflection of ∼0.69mm , which exceeds the design criteria. Set 4 has a total deformation around 0.42mm with an eigenvalue buckling of ∼45 , which is a good safety factor. As Set four provides the maximum mass reduction among Set 1–4 with deflections within the desired limits, it is considered as the reference for design modifications. The idea is similar to that seen in *End-over-end walking robot*, i.e., to keep the ends wider with the middle link section slimmer. The updated design and FEA simulation results are depicted in Figure 13A.

**TABLE 10:** Comparison of design parameters for different link dimensions.

Set	Outer dia ( mm )	Thickness ( mm )	Mass ( kg )	BM ( Nm )	Stress ( MPa )	Max deflection ( mm )	Load multiplier
Set 1	50	3.5	0.149	34.1	22.88	0.215	61.548
Set 2	45	3.5	0.132	34.03	29.12	0.284	58.268
Set 3	40	3.5	0.118	33.9	36.841	0.328	53.916
Set 4	35	3.5	0.103	33.86	44.841	0.424	44.805
Set 5	30	3.5	0.087	33.8	59.368	0.688	39.051

### Base plate design analysis

E-Walker’s prototype has a base plate equipped with on-off electromagnets on either end to fix itself on metallic structures and carry payloads (Electromagnet 2022). The maximum load on the base plate would be experienced when the E-Walker is erectly carrying the payload ( ∼2kg ). The base plate would then have to bear the weight of the seven actuators, clamps, two links and the payload. A cumulative normal force of 150N was applied on the base plate’s top face. A torque of 100N was also applied to take into consideration any moments generated. Figure 13B presents the SSA results illustrating a deformation of 0.002mm and a load multiplier of ∼4000 , which is a sufficient safety factor.

## Mission concept of operations

**FIGURE 14:**
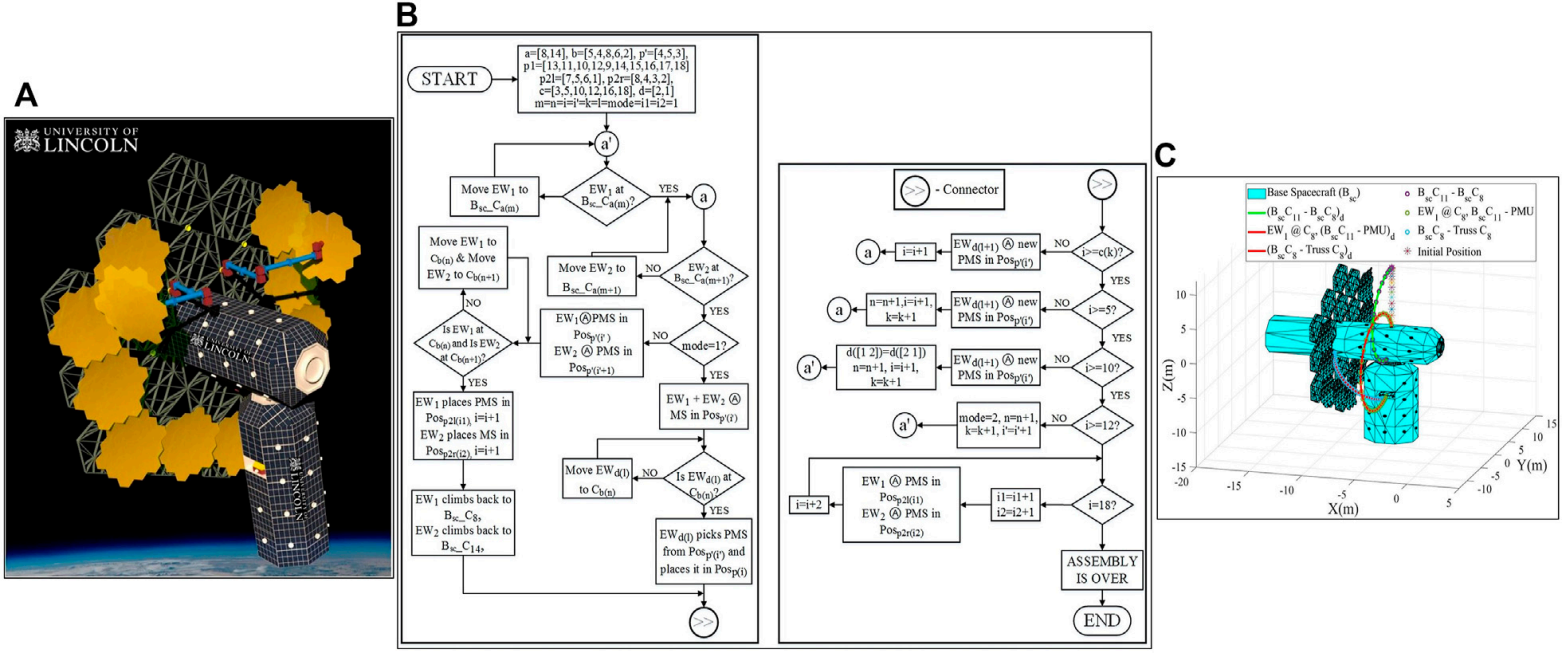
Mission scenario 2b: **(A)** Artistic illustration **(B)** flow chart **(C)** simulated trajectory.

A detailed feasibility analysis was carried out in a previous publication (Nair et al., 2020b), where a potential mission scenario, namely Mission Scenario 2b, was shortlisted after a trade-off analysis of eleven mission scenarios. A maximum of four E-Walkers were considered to carry out the assembly of the 25 m LAST. The analysis took into consideration the mission cost, lifting mass, net power requirements, control and motion planning complexity. This paper presents Mission Scenario 2b, which involves only two E-Walkers, namely EW_1_ and EW_2_ to carry out the in-orbit assembly of the 25 m LAST (Figure 14A). The mission ConOps provide an insight into task-sharing and co-manipulation with the collaborative operation between the two E-Walkers. This mission concept facilitated by two E-Walkers could facilitate futuristic LAST assembly missions with much larger apertures of 50 m or 100 m.

Mission Scenario 2b involves both the E-Walkers sharing equal responsibilities in the assembly process. The tasks are split into assembling the PMS using the PMUs and performing pick-and-place operations to place the assembled PMSs to assemble the 25 m PM. To undertake the assembly, each of the E-Walkers is initially assigned one-half of the truss. This paper considers EW_1_ to take up the responsibility of the left half of the truss, whereas EW_2_ is responsible for the right half. The E-Walker’s reach is considered large enough to attend to the inner-ring assembly (positions 1–6) by staying on the B_sc_. For the outer-ring assembly (positions 7–18), the E-Walkers will move to pre-designed connector points on the truss.

For a quick and efficient assembly, the operation is carried out in two modes. In Mode 1, both the E-Walkers collaborate to assemble a PMS, using the PMUs stored in the S_sc_, in the inner ring of the truss. Once a PMS is assembled, one E-Walker steps onto the respective connector point on the truss (EW_1_ for the left-half and EW_2_ for the right-half) to pick and place the assembled PMS onto the respective outer-ring positions. Considering an operation where EW_1_ moves onto a left-half connector point on the truss to place a PMS, EW_2_ will begin assembling a new PMS while EW_1_ is away. Once EW_1_ places the PMS, it climbs back onto the B_sc_ to assist EW_2_ with the ongoing PMS assembly. For the right-half assembly, the roles of the E-Walkers are switched. Mode 1 of operation sees a majority of the outer ring being assembled.

Mode two of the assembly operation involves both the E-Walkers taking up individual responsibilities to assemble the PMS and to perform the pick-and-place operation in their respective halves. This is with the intent to utilise the E-Walker’s walking capability and reduce the overall sizing of the E-Walker. Additionally, each of the E-Walker will assemble PMSs simultaneously in the inner ring. The E-Walkers can attend to their respective halves in the inner ring from the B_sc_ itself. In Mode 2, both the E-Walkers collaborate to work independently and simultaneously, resulting in a faster assembly.

### Assembly constraints and strategy

The LAST mission poses a few constraints to the E-Walker for an efficient assembly. For example, the E-Walker should maintain a clearance of 0.5–1 m from the assembled PMSs to reduce the chances of any collision. Additionally, while assembling or placing the PMSs, the E-Walker should make sure the task is not being carried out in an in-zone mirror position. Therefore, it is understood that the E-Walker has to be outside the zone of assembly at all times. A few strategies for assembly that takes into account these constraints are:

1) The assembly process can begin with the E-Walkers assembling the PMSs using the PMUs stored in the S_sc_. Realising the fact that the E-Walkers have a reach to assemble the inner-ring positions from the B_sc_, the assembly of the PMSs can be initially conducted on a certain inner-ring position. This allows the E-Walkers to walk around freely between the B_sc_ and truss.
2) The outer-ring positions can be filled in first. The assembled PMS in the inner ring can then be placed on to an outer ring position by an E-Walker moving onto the respective connector point based on their assigned halves. Assemble the outer ring mirrors first to allow the E-Walker to walk around the truss during the assembly process.
3) The mode of operation can be switched when a situation arises i.e., when the independent task execution of the E-Walkers in their assigned halves can help with a quick and efficient assembly.
4) Once the outer ring is completely assembled, the E-Walkers can climb back onto the B_sc_ to fill in the inner ring positions.

### Flow chart of assembly process (mission scenario 2b)

The flowchart uses the following abbreviations:

B_sc_ - Base Spacecraft; B_sc__C - Base Spacecraft Connector; C_b_ - Connector point on truss; Ⓐ - assembles/assembling; PMS - Primary Mirror Segment; EW- End-Over-End Walker; Pos - Position.

The mirror positions with their inner and outer ring positions and the corresponding connector points on the truss can be recalled from Figure 3. The connector point numberings on the B_sc_ can also be understood from Figure 3. With the information on Mission Scenario 2b provided in *Mission concept of operations (ConOps)*, a flowchart is presented in Figure 14B, which provides more in-depth insight into the mission ConOps. The terms used are:

a—In use Connector number array on B_sc_; b - In use Connector number array on Truss.
mode—select the mode of operation.
mode 1—where EW1 and EW2 work together.
mode 2—EW1 and EW2 work parallelly on the left half and right half mirrors. p_2l_ and p_2r_ mirror arrays would be assembled in mode 2.
p'—Array of Initial Mirror position on Truss
p_1_—Array of outer-ring mirror positions to be assembled during mode 1
p_2l_—Array of final mirror positions to be assembled by EW_1_ on left-half of the truss–mode 2.
p_2r_—Array of final mirror positions to be assembled by EW_2_ on right-half of the truss (Positions in sequence: 8, 4, 3, 2)—mode 2.
c—PMS counter; m—array index of a; n—array index of b; i'—array index of p'; k—array index of c;
i—array index of p, provides insight into the number of assembled PMS.

It is to be noted that the above-mentioned variables m, n, i, i' and k start from 1.

d—Array of E-Walker Numberings; m - array index of a; n - array index of b; i_1_—array index of p_2l_
i_2_—Array index of p_2r_; l—Array index of d

### Mission scenario 2b analysis

The assembly process of the 25 m LAST in the flowchart for Mission Scenario 2b begins with EW_1_ and EW_2_ taking their positions on their B_sc_ connector ports. EW_1_ takes a position on B_sc__C_8_ and EW_2_ on B_sc__C_14_. Positioning the E-Walkers on two sides of the B_sc_ helps with their move onto the truss connector ports in the respective halves. Now, the truss positions can be filled in with the assembled PMS from either half of the truss. In this paper, the assembly of the right-half outer ring of the truss is considered first. The positions of the truss are filled in a specific sequence based on the positions shown in Figure 2. The mission has two modes of operation to carry out the assembly.

The mode is initially set to 1 suggesting the collaborative working of both the E-Walkers. During mode 1, both the E-Walkers begin assembling the PMS on the inner-ring truss position 4, using the PMUs stored in the S_sc_. Throughout the operation, the PMUs are considered to be readily available. Once the first PMS is assembled, EW_2_ moves onto C_5_ on the truss. EW_2_ then picks the assembled PMS from position four and places it on position 13, filling the first position of the 25 m LAST. While EW_2_ is performing the pick-and-place operation, EW_1_ starts assembling the second PMS. Post placing the first PMS, EW_1_ moves onto the B_sc_ to assist EW_1_ with the assembly of the second PMS. Positions 11 and 10 are filled similarly as seen with the first PMS, completing the placement of three PMSs. Thereafter, the same process is repeated to fill in positions 12 and 9, with EW_2_ moving onto C_4_ as position 12 is in-zone of C_5_. Now, a majority of the right-half outer ring is filled in.

To assist with the assembly of the left-half outer ring, the roles of the E-Walkers are switched. EW_1_ and EW_2_ assist with the assembly of the PMS, while EW_1_ now has an added responsibility to place the assembled PMSs by moving onto C_8_ on the truss. C_8_ helps the EW_1_ fill positions 14, 15, 16, 17 and 18 to successfully complete the placement of ten PMSs. Similar to the right-half assembly, EW_2_ continues to assemble a new PMS while EW_1_ is placing the previously assembled PMS. Once the assembled PMS is placed, EW_1_ climbs back onto the B_sc_ to assist EW_2_ with the assembly of the new PMS.

The mode of operation now switches to mode 2. With eight positions left to be filled, two positions are in the outer ring, while six positions are in the inner ring. In mode 2, EW_1_ and EW_2_ have individual responsibilities of assembling the PMS and perform the pick-and-place operation in their respective halves. To fill out the outer ring, EW_1_ assembles a PMS in inner-ring position five while EW_2_ assembles a PMS in position 3. Post assembling the PMS, EW_1_ moves onto C_6_ and EW_2_ to C_2_ on the truss. EW_1_ moves the assembled PMS from position five to position 7, while EW_2_ places the PMS on position 8. This step is well represented in Figure 14A. This marks the successful assembly of the outer ring of the 25 m PM. For the inner-ring assembly, EW_1_ and EW_2_ move onto B_sc_ and performs a simultaneous assembly of the PMSs on each half of the truss. While EW_1_ assembles the inner ring positions 5, 6 and 1, EW_2_ completes the assembly by filling up positions 4, three and 2. The assembly of the 18 PMSs marks the successful completion of the 25 m LAST.

The systematic assembly presented eliminates the risks of the E-Walker colliding with the mirror modules by avoiding in-zone mirror positions. This helps in satisfying the top-level requirement R4 of the E-Walker. Requirements $R_{2}$ , $R_{3}$ , $R_{4}$ and $R_{5}$ are to be achieved throughout the mission, to fulfil requirement $R_{1}$ . It is to be noted that in this paper the successful tracking of the joint parameters at each step by avoiding in-zone mirror positions is assumed to result in a collision-free assembly. However, in a real mission scenario there exists possibilities of the E-Walker colliding with the B_sc_, S_sc_ and Truss due to inappropriate docking. Successful docking is achieved with the help of Camera Lighting Units on the E-Walker alongside the controller constantly tracking the joint parameters under perturbed conditions.

### Mission trajectory tracking

Figure 14C shows the trajectory tracking of EW_1_ during the Mission Scenario 2b assembly process in a simulated environment. Trajectory 1 represents EW_1_’s motion from B_sc__C_11_ to B_sc__C_8_. The motion is quite similar to Phase-1 of Cycle-1. Considering joint 1 is fixed at B_sc__C_11_, the motion of the EE attached to q_7_ is tracked to reach B_sc__C_8_. This satisfies the initial condition check for EW_1_ in the assembly process, depicted in the flow chart. Trajectory two represents the tracking of joint 1’s motion to fetch a PMU from the S_sc_. During this motion, joint five is locked at B_sc__C_8_. The third trajectory portrays a common motion for EW_1_ during the operation in Mode 1, where EW_1_, with joint five fixed to B_sc__C_8_ moves onto C_8_, on the truss, to pick and place the assembled PMS in the relevant truss position on the left half of the truss.

## Conclusion and future research

This paper introduced the design and feasibility of a walking space manipulator for *in-situ* robotic assembly of a 25m Large Aperture Space Telescope (LAST). It captured the critical challenges of in-space robotic assembly mission, elicited the mission and system-level requirements and provided a deeper understanding of the design engineering of the E-Walker. The seven DoF End-over-End Walking Robot (E-Walker) selected for this mission provides the modularity, dexterity features alongside manoeuvring capabilities over an enhanced workspace. Detailed design engineering helped identify an optimised design for the E-Walker to carry out the assembly of LAST. These included static structural and modal analysis of the full-scale space design of the E-Walker. This provided an insight into understanding the dynamical behaviour of the intercoupled links and the end-effectors of the E-Walker while carrying a payload under different environmental conditions.

The E-Walker presented would be able to extend the mission lifecycle by carrying out routine maintenance and servicing missions post assembly. The E-Walker design shown is versatile, and it is a candidate robot for future planetary and orbital missions. In addition to the full-scale design, a scaled-down ( 1:6 ) prototype design of the E-Walker is also presented, along with its detailed structural analysis. The optimised design analysis (considering a payload) identifies the E-Walker prototype to also be an ideal candidate for servicing, maintenance, and assembly operations on the ground. An example could be the E-Walker carrying out regular maintenance checks on a wind turbine, given its capability to manoeuvre anywhere in a 3D space. Once the detailed design engineering for the E-Walker was discussed, a mission concept, namely Mission Scenario 2b, was presented to carry out the assembly of the 25 m LAST. The assembly flow presented using the dual-agent system (two E-Walkers) realises robotic task-sharing capabilities in orbit. The mission serves as a baseline study for large-scale future missions requiring multiple agents. Overall, this paper presents the top-level system design to assemble a 25 m telescope in orbit, with a detailed analysis of the E-Walker design to be integrated into future in-orbit and terrestrial operations. This paper is a useful case study for future LAST missions with Primary Mirrors of apertures planned up to 100m .

Future research includes finalising the design specifications of the true-scale E-Walker for the 25 m LAST mission. Additionally, an Earth-based small-scale E-Walker prototype is under development for further hardware-in-loop experimentation. This prototype can then be tested with space-qualified robust controllers, like the Proportional-Integral-Derivative-Computed-Torque-Controller or the H_∞_ controller for precise joint tracking under external disturbances and parametric uncertainties. Given the huge commercial interest in this topic, Space agencies and industries have developed their road maps for orbital robotics missions. The LAST mission and the E-Walker model have also drawn commercial and scientific attention. Over the next 5–10 years, it is envisaged that researchers and industries will carry out further development to assess E-Walker’s feasibility. These include prototyping an E-Walker for a precursor mission involving a 2.5m/5 m LAST. The E-Walker’s capability for assembling the secondary mirror, truss modules and baffles is not yet realised.

## Data Availability

The original contributions presented in the study are included in the article/Supplementary Material, further inquiries can be directed to the corresponding author.
